# Therapeutic Strategies for High-Dose Vasopressor-Dependent Shock

**DOI:** 10.1155/2013/654708

**Published:** 2013-09-15

**Authors:** Estevão Bassi, Marcelo Park, Luciano Cesar Pontes Azevedo

**Affiliations:** ^1^Intensive Care Unit, Discipline of General Surgery and Trauma, Hospital das Clínicas da Faculdade de Medicina da Universidade de São Paulo (USP), Avenue Eneas de Carvalho Aguiar 255, 4th Floor, 05403-000 São Paulo, SP, Brazil; ^2^Intensive Care Unit, Emergency Medicine Discipline, Hospital das Clínicas da Faculdade de Medicina da Universidade de São Paulo (USP), Avenue Eneas de Carvalho Aguiar 255, Room 5023, 05403-000 São Paulo, SP, Brazil; ^3^Intensive Care Experimental Laboratory, Research and Education Institute, Hospital Sirio-Libanes, Rua Cel. Nicolau dos Santos, 69 01308-060 São Paulo, SP, Brazil; ^4^Intensive Care Unit, Federal University of São Paulo (UNIFESP), Rua Napoleão de Barros 715, 04024002 São Paulo, SP, Brazil

## Abstract

There is no consensual definition of refractory shock. The use of more than 0.5 mcg/kg/min of norepinephrine or epinephrine to maintain target blood pressure is often used in clinical trials as a threshold. Nearly 6% of critically ill patients will develop refractory shock, which accounts for 18% of deaths in intensive care unit. Mortality rates are usually greater than 50%. The assessment of fluid responsiveness and cardiac function can help to guide therapy, and inotropes may be used if hypoperfusion signs persist after initial resuscitation. Arginine vasopressin is frequently used in refractory shock, although definite evidence to support this practice is still missing. Its associations with corticosteroids improved outcome in observational studies and are therefore promising alternatives. Other rescue therapies such as terlipressin, methylene blue, and high-volume isovolemic hemofiltration await more evidence before use in routine practice.

## 1. Introduction

In-hospital mortality of circulatory shock requiring vasopressors exceeds 50% and nearly 40% of these deaths are caused by progressive hypotension despite support [1]. There is no consensual definition of refractory shock. Increasing doses of vasopressors are associated with unfavorable outcomes [2] and there is a wide range of cut-offs used to identify doses associated with higher mortality, including 15 to 100 micrograms per minute of norepinephrine (NE), for example [3, 4].

High-dose vasopressor-dependent shock is often seen as a terminal event in the intensive care unit. On one hand, it is commonly argued as futile to administer high-dose vasopressors in the critically ill patient with multiple organ failure [5]. On the other hand, survival is up to 50% in “severe” septic shock patients receiving early treatment with a specific algorithm [6].

 Unfortunately, high-quality data to guide therapy in this situation are scarce. While several rescue strategies were described, few studies compared them. The objective of this narrative review is to summarize part of this evidence to help clinicians in the management of this extreme condition.

## 2. Definition and Epidemiology

There is no consensual definition of refractory shock, and many cut-offs were used in diverse clinical scenarios (Table 1). Norepinephrine (NE) doses > 0.5 mcg/kg/min or need for rescue therapy with vasopressin generally is associated with mortality rates higher than 50%, while 94% of patients requiring concentrations above 100 mcg/min of NE or epinephrine died in one study [4].

 The threshold of approximately 0.5 mcg/kg/min of NE is often used in clinical trials as a definition of refractory shock [8, 9, 13]. In 2011, Benbenishty et al. used a ROC analysis to determine the correlation between maximum dose of vasopressors and death. The area under the curve was high (0.85), and the administration of concentrations above 0.5 mcg/kg/minute of  NE or epinephrine demonstrated sensitivity of 96% and a specificity of 76% for the likelihood of mortality [2]. Interestingly, the same dose was the inflexion point of the mortality curve in the study by Luckner et al. [14]. In the recent study by Brown et al., high-dose vasopressor therapy was defined as use of more than 1 mcg/kg/min of norepinephrine equivalents [12]. Mortality at 90 days was 83%, which may suggest that rescue therapies could be considered earlier in the evolution of shock. 

 Regarding the incidence of refractory shock, Mayr et al. described the causes of death and outcomes of critically ill patients and attributed 17.8% of deaths to refractory cardiovascular failure, while the main cause of death in the ICU was acute multiple organ failure [16]. In the recent randomized SOAP 2 study including 1,679 patients with shock from diverse etiologies, 43% of deaths were due to refractory shock [1]. Kumar et al. attributed 55% of deaths to this entity in their retrospective analysis of 4,662 patients with septic shock, a finding similar to the SOAP 2 one [17]. However, a clear limitation of this type of analysis is the inclusion only of non-survivors. Thus, some efforts to specifically evaluate the epidemiology of refractory shock were done. In the study by Benbenishty et al., 7% of patients required >0.5 mcg/kg/minute NE or epinephrine during ICU stay [2]. Jenkins et al. found that 6% of their ICU patients required concentrations above 100 mcg/min of NE or epinephrine [4]. Despite the difference in dosages, taking together these data suggest that approximately 6-7% of critically ill patients will develop refractory shock.

## 3. Etiology

Every cause of acute cardiovascular failure can progress to refractory shock. Since mortality in refractory shock can be as high as 94% [4], efforts should be made to find the cause(s) for the patient's syndrome.

 Clinicians should specifically search for potential reversible causes. Hypovolemia should be ruled out, by means of a fluid challenge or dynamic maneuvers, such as the passive leg-raising test with cardiac index measurement [18]. The diagnosis of pericardial tamponade can be misleading, and a low degree of suspicion should trigger appropriate investigation since pericardiocentesis can be lifesaving in this setting [19]. In the extreme clinical scenario of refractory cardiovascular failure, the etiology and specific treatment of shock should be aggressively pursued in every cause of shock, including sepsis [20], myocardium ischemia complicated by cardiogenic shock [21], and massive pulmonary embolism [22]. In this context, exclusive supportive treatment will probably fail. 

## 4. Pathophysiology

Distributive shock is an important component in virtually all forms of advanced shock [23]. Even when the initial shock is not vasodilatory *per se*, the systemic hypoperfusion triggers an inflammatory response that leads to inappropriate vasodilatation and persistent hypotension [24, 25]. Numerous mechanisms contribute to vasodilatory shock in inflammatory states, including cytokine-induced increased expression of inducible nitric oxide synthase (iNOS). This enzyme produces nitric oxide (NO), a potent endogenous vasodilator, in high concentrations. Several other stimuli present in shock, like cellular hypoxia, acidosis and, NO itself, activate ATP-sensitive potassium channels. These channels induce membrane hyperpolarization, which prevents an increase in the cytoplasmic calcium, leading to vasodilatation. Other mechanisms contribute to the pathophysiology of inflammatory shock, such as critical illness-related corticosteroid insufficiency, inappropriately low plasma levels of vasopressin, oxidation, and inactivation of catecholamines. Altogether these mechanisms lead to loss of vascular tone and hyporesponsiveness to vasopressors [23, 26], which are the main characteristics of refractory shock.

 On the other hand, it is notorious that cardiac dysfunction due to inflammation may be present in other shock states such as septic [27] and hemorrhagic [28]. This reinforces the typical clinical picture seen in advanced shock: a patient with one clear shock etiology but with multiple components contributing to refractory circulatory collapse.

## 5. Management of Refractory Shock

### 5.1. Monitoring

 Since many mechanisms can contribute to circulatory collapse, hemodynamic assessment is crucial in the management of refractory shock. It is not clear the best method to monitor these patients, but it is important to use tools that evaluate volume status and cardiac function. 

 Refractory shock represents an extreme failure of cardiovascular system, with high short-term mortality ratios. In this setting, it seems appropriate to guarantee adequate fluid resuscitation, while minimizing its side effects since endothelial dysfunction present in SIRS can produce only transient responses after volume expansion. This is probably better achieved by identifying patients that will improve hemodynamically after fluid *bolus*, avoiding deleterious consequences of hypervolemia.

 It is very difficult to assess fluid responsiveness based only on clinical signs or static parameters [29]. Cardiac output can be measured before and after volume expansion, by thermodilution or less invasive methods [30]. In most clinical trials, an elevation >15% of cardiac output after 500 mL volume infusion is considered positive fluid responsiveness. Alternatively, the use of dynamic parameters such as pulse pressure variation [29], passive leg raising test with cardiac index measurement [18], inferior vena cava diameter variation [31] or arterial waveform derived variables [32] can predict which patients will increase cardiac output after volume expansion. A negative test in these maneuvers or failure in the improvement of cardiac index by fluid *bolus* should encourage clinicians to stop volume expansion, even in the context of refractory shock. Regarding the type of fluids for resuscitation, the recent Surviving Sepsis Campaign Guidelines 2012 recommend crystalloids to be used as the initial fluid of choice in the resuscitation of septic patients and against the use of synthetic colloids, specifically hydroxyethyl starches (HES) for this purpose based on the results of recent clinical trials [33]. 

 Urine output, capillary refill time, assessment of peripheral perfusion, superior vena cava oxygenation saturation (ScvO_2_), or mixed venous oxygen saturation (SvO_2_) and lactate concentrations should be evaluated as markers of tissue hypoperfusion in every patient with shock. The goals of resuscitation are urine output ≥ 0.5 mL/kg/hr, ScvO_2_ > 70% or SvO_2_ > 65% and to decrease lactate levels ≥20% every 2 hours if these concentrations were initially increased. After adequate fluid resuscitation and stabilization of arterial pressure with vasopressors, if low ScvO_2_/SvO_2_ or high lactate levels persist, additional efforts to improve tissue oxygenation should be made. Alternative approaches for this scenario include transfusion of packed red blood cells if anemia is present (hematocrit < 30%) or inotrope infusion [33–35], despite controversies associated with these interventions.

In these severely ill patients with refractory shock and ongoing signals of systemic hypoperfusion, tools to monitor cardiac function may help to guide therapy, even though this option remains debatable [36, 37]. These tools may include echocardiography, pulmonary artery catheter (PAC), and minimally invasive monitoring devices. In the recent years there has been a significant decrease in the utilization of PAC following clinical trials with negative results. It is important to emphasize, however, that these studies were not conducted specifically in refractory shock patients, which represent a situation with elevated short-term mortality rates due to circulatory failure. The use of minimally invasive or non-invasive techniques (e.g., pulse contour methods, and echocardiography) to monitor cardiac output has the advantage of avoiding risks associated with pulmonary artery catheterization. However, the accuracy of pulse-contour devices may be compromised by periods of arrhythmias and significant vasoplegia, which may be a serious issue in refractory shock. In addition, specific ventilatory parameters are required to improve the measurements, which are not commonly used in these patients. While reliable tracking of changes in cardiac output and other hemodynamic variables in critically ill patients seems more important than accuracy *per se*, that approach was not adequately evaluated in large clinical trials [38–40]. Bedside-focused cardiac ultrasound has several advantages that include its noninvasive nature and the ability to provide information about differential mechanisms and physiology contributing to ongoing shock (e.g., hypovolemia, and myocardial dysfunction). However, it requires training and it is not a continuous method (though it can be repeated as necessary) [41]. Thus, we suggest using one of the above methods of hemodynamic monitoring to help guide therapy in refractory shock patients especially in the subgroup with persistent signs of hypoperfusion after fluid resuscitation and stabilization of arterial pressure with vasopressors. 

### 5.2. Corticosteroids

 The use of steroids in septic shock has been controversial for many years. Studies with anti-inflammatory high dose of corticosteroids were conducted until the 1980s and as a whole demonstrated no benefit [42]. Since the 1990s the concept of relative adrenal insufficiency encouraged the use of supraphysiologic low dose of steroids in sepsis, and large randomized studies were conducted, although there is, as yet, no definitive answer. 

 There is evidence that steroids improve hemodynamic stability and decrease need for vasopressors in patients with septic shock [42–44]. Annane et al. demonstrated that the response to norepinephrine is improved one hour after a 50 mg *bolus* of hydrocortisone [43], indicating rapid onset of action. Recent data corroborate that the effect of corticosteroids in hemodynamics is predominantly mediated by vascular tone, independent of adrenal function tests [42, 45]. However, the adverse effects of steroids use including superinfection, hypernatremia, and hyperglycemia can occur even with “stress dose” hydrocortisone (200–300 mg/day) [42], and this may help to explain the lack of benefit seen in the CORTICUS trial [45]. This study was the largest multicenter, randomized recent trial of low dose hydrocortisone (50 mg 6/6 h) in septic shock patients. There was no clinical benefit of the drug and the corticotrophin test was not useful as prognostic marker or as screening tool for patients who would benefit from such therapy [45]. 

 These findings were in contrast with those previously reported by Annane et al. in 2002 [44]. In this prospective randomized trial, patients received either hydrocortisone (50 mg every 6 hours) and fludrocortisone (50 *μ*g once daily) or matching placebo for 7 days. There was improved global 28-day survival, especially in nonresponders to the corticotropin test. The differences in outcomes between the two trials are attributed, to a greater extent, to different patient populations [42]. In CORTICUS trial, patients were less severely ill, as underlined by lower SAPS II score. Hemodynamic criteria for study entry were also different. In CORTICUS, patients had shock defined by systolic blood pressure of <90 mmHg despite adequate fluid replacement or need for vasopressors. In Annane et al. trial, inclusion criteria include hypotension despite adequate fluid replacement and vasopressor support. The analysis of baseline characteristics suggests that patients in CORTICUS were in use of lower doses of vasoactive agents, despite not being hypotensive, while patients had a mean arterial pressure of 55 mmHg in Annane et al. trial [44, 45]. In this setting, refractory shock patients are probably better represented by Annane trial than by CORTICUS.

 In a recent study specifically evaluating refractory shock, Brown et al. described 443 patients requiring more than 1 mcg/kg/min of norepinephrine equivalent, and in their trial stress-dose corticosteroid therapy was a protective factor for mortality [12].

 Another source of controversy is the lack of use of mineralocorticoids in the intervention group of CORTICUS trial. This question was specifically addressed in the COIITSS study. In this trial, 509 septic shock patients with sequential organ failure assessment score of ≥8 who received stress-dose hydrocortisone were randomized in a 2 × 2 factorial design to intensive versus conventional glycemic control and to receive 50 mcg of fludrocortisone versus hydrocortisone alone. There was no statistically significant improvement of in-hospital mortality with the addition of oral fludrocortisone in this population [46]. Despite some methodological problems, as the lack of placebo versus fludrocortisone comparison, this is probably the best recent available evidence to guide fludrocortisone use in septic shock.

 Systemic inflammatory response syndrome (SIRS) with exacerbated vasodilatation also contributes to hyporesponsiveness to vasoactive agents in shock of nonseptic origin. Hoen et al. demonstrated that hydrocortisone increases vasopressor response to phenylephrine following severe trauma, suggesting a role for steroid therapy in ameliorating shock following hemorrhagic shock [47]. Small studies with stress-dose hydrocortisone were also done in cardiac surgery [48] and burn [49] patients, with short-term benefit. 

 In summary, low-dose hydrocortisone (50 mg intravenous bolus every 6 hours) may be of benefit in refractory septic shock patients, which were better represented by Annane et al. study than by CORTICUS trial. The use of adjunct fludrocortisone seems unnecessary, in view of the COIITSS clinical data [46]. SIRS contributes to refractory shock from non-septic etiologies, and stress-dose steroids could play a role in this setting, although more clinical trials are needed.

### 5.3. Arginine Vasopressin

 Relative arginine vasopressin (AVP) deficiency is common in vasodilatory shock [50], and exogenous infusion is often used as rescue therapy in refractory shock [13–15]. In 2003, Dünser et al. specifically addressed the question whether there was benefit from adding AVP 0.067 U/min in hypotensive patients requiring NE > 0.5 mg/kg/minute in a randomized trial including 48 patients with vasodilatory shock. AVP improved physiological variables such as gastric tonometry, and there was a lower incidence of tachyarrhythmias in AVP-treated patients, but no clinical benefit could be found due to the small sample size [8].

 The VASST trial was a multicenter, double blind trial that randomized 778 patients with septic shock to receive AVP (0.01 to 0.03 U per minute) or norepinephrine in addition to open label vasopressors. Overall, there was no benefit of AVP infusion in 28 or 90-day mortality. The rate of serious adverse events was also similar between groups, including arrhythmias and mesenteric ischemia. In the subgroup of patients with less severe septic shock (*a priori* defined by baseline norepinephrine dosing <15 *μ*g/min), an improved survival with vasopressin was noted. This finding was not observed in patients with more severe shock [3].

 Although VASST trial was not designed to specifically study vasopressin as a rescue therapy in refractory shock, it is the largest randomized trial comparing AVP with catecholamines. Even though other studies tested higher doses of vasopressin (up to 0.067 IU/min) [36], more data are necessary before advocating these doses not tested in VASST (up to 0.03 IU/min) in clinical practice, since a large number of patients are required to detect difference in adverse effects.

 Data derived from VASST compared hemodynamic profile of vasopressin versus norepinephrine infusion. There was a significant reduction in heart rate with AVP but no change in cardiac output. Despite this, a greater use of inotropic was needed with vasopressin, particularly in the more severe shock stratum, in which the lack of benefit from AVP infusion was clearer. This finding suggests caution when using AVP as rescue therapy in patients with refractory shock, particularly in those at risk for cardiac dysfunction, since inotrope infusion may be necessary to maintain cardiac output [51]. 

 Interaction of vasopressin infusion and corticosteroid treatment, both frequently used to treat refractory shock, was also analyzed in VASST population in a post-hoc study. In patients who received steroid therapy, vasopressin was associated with decreased mortality. Interestingly, in patients who did not receive corticosteroids, AVP compared to NE group had increased mortality. Steroids use also increased plasma vasopressin levels by 33% [52]. Similar findings were described in retrospective studies in different populations [12, 13]. While such interaction could be a good alternative to treat refractory shock, randomized trials are necessary to validate this hypothesis.

### 5.4. Terlipressin

Terlipressin (TP) is a synthetic analog with theoretical advantages over AVP, such as longer half-life (which could avoid rebound effect) and higher selectivity for V1 receptor (which could produce more potent vasoconstriction with less adverse effects) [53]. 

 In 2005, Albanèse et al. randomized patients with hyperdynamic septic shock to receive NE or a *bolus* of 1 mg of TP. The *bolus* could be repeated if hypotension recurred during the 6 h study period. Though both drugs increased mean arterial pressure, TP decreased heart rate, cardiac index and oxygen consumption [54]. 

 The DOBUPRESS trial demonstrated that inotrope infusion could counterbalance these adverse cardiac effects of TP; however, a mean dose of 20 *μ*g/kg/min of dobutamine was necessary to reverse terlipressin-induced decrease in SvO_2_. The benefit of such therapy is questionable since patients may be exposed to adverse effects of both drugs [55]. 

Another option to prevent these deleterious cardiac effects of TP was tested in ovine endotoxemia. In this study, intermittent *bolus* of terlipressin induced acute decreases in heart rate and cardiac index and increases in pulmonary vascular resistance, effects that were probably linked to those seen in clinical trials. Continuous infusion of the drug could prevent those adverse effects with lower cumulative dose, leading to a new possibility for TP administration [56].

 The TERLIVAP study randomized 45 septic shock patients to receive continuous infusion of terlipressin (1.3 *μ*g/kg/h), vasopressin (0.03 U/min), or norepinephrine associated with open label norepinephrine to achieve target mean arterial pressure. There was no significant hemodynamic difference among groups, suggesting that continuous infusion of TP is probably a safer way to administer this drug in clinical situations [57]. 

 In summary, clinical trials testing TP in septic patients were not designed in the context of refractory shock. Evidence is scarce, but if TP is chosen as rescue therapy in refractory shock, continuous infusion (1.3 *μ*g/kg/h) is probably safer than intermittent infusion.

### 5.5. Nitric Oxide Inhibitors

 Since nitric oxide contributes to inflammatory vasodilatation and cardiac dysfunction, large prospective randomized studies with nitric oxide inhibitors were done in septic and cardiogenic shock [58, 59].

 In 2004, a multicenter randomized controlled trial randomized 797 patients with septic shock to receive nitric oxide synthase (NOS) inhibitor 546C88 or placebo. The trial was stopped early for increased 28-day mortality in intervention group (59% versus 49%). Most of the excess mortality with 546C88 was attributed to cardiovascular failure, possibly due to exaggerated vasoconstriction with the study drug [58]. 

 The TRIUMPH trial randomized 398 patients with cardiogenic shock to NOS inhibition with tilarginine or matching placebo. Despite increased arterial pressure with NOS inhibition, there was no clinical benefit and the trial was stopped early for futility [59]. 

 Since nitric oxide has both deleterious and beneficial effects in inflammation, maybe these disappointing results were not so surprising at all. As an example, nitric oxide reduces platelet aggregation and increases macrophage activity in sepsis [60]. In this context, methylene blue (MB), which targets a downstream pathway in vasodilatation via guanylate cyclase, may be a better option, by preserving other actions of NO. Despite being used for a long time as an adjuvant therapy, adequate controlled trials using this drug in shock are scarce. 

 In 2001, Kirov et al. randomized 20 patients with septic shock to receive isotonic saline or methylene blue (2 mg/kg *bolus* followed by stepwise continuous infusion for 4 hours). MB reduced vasopressor requirements and prevented decrease in cardiac function due to sepsis. Contrary to previous studies, there was no detrimental effect on pulmonary gas exchange. The trial was not powered to assess clinical outcomes, but there was a trend towards improved shock resolution with MB [61].

 Nitric oxide is also a mediator of SIRS and vasoplegia after cardiac surgery. In 2004, Levin et al. randomized 56 patients with vasoplegia to receive 1.5 mg/kg in 1 hour of MB or placebo. MB reduced the duration of vasoplegia and improved mortality in these patients (0 versus 21%) [62].

 Optimal dosing for MB in shock is unknown. Most studies used a* bolus* dose of 1-2 mg/kg. Doses higher than 3 mg/kg can compromise splanchnic perfusion [63] and should be avoided. An initial dose of 2 mg/kg followed by continuous infusion of 0.25 mg/kg/hour is another option [60]. The effects of MB in prolonged infusion (e.g., >24 h) were not adequately studied.

 Most studies with MB in shock were observational, and the therapy was initiated very late in the course of shock, when the mortality is very high and there are few chances of clinical improvement [60]. Maybe lower thresholds (as an example 0.5 mcg/kg/min of NE) for testing rescue therapies as MB in refractory shock will provide better evidence to guide treatment in this severe condition.

 At this moment, given the results of largest trials with nitric oxide pathway inhibitors [58, 59], the use of these drugs in refractory shock deserves more studies before clinical application. 

### 5.6. Inotropes

Inotropes are frequently used in refractory shock when a hypodynamic hemodynamic pattern is present. Castro et al. advocate a resuscitation algorithm for severe septic shock (defined as NE requirements >0.3 *μ*g/kg/min), which includes epinephrine use if cardiac index is below 3 L/min/m^2^ [6].

 The use of epinephrine instead of dobutamine as an inotropic agent in refractory shock has the theoretical advantage of providing adjunct vasoconstriction with *β*-stimulation, minimizing the risk of vasodilatation with consequent hypotension. On the other hand, with the concomitant use of dobutamine and NE, clinicians can separate both *α* and *β* effects of vasoactive agents, with more control of these actions [64].

 The CATS study was the largest trial comparing these two strategies. In this study, 330 patients were randomized to receive epinephrine or norepinephrine plus dobutamine, with no significant difference in outcomes or serious adverse events between the groups. Nevertheless, it is important to remind that epinephrine was associated with delay in lactate clearance, probably due to exaggerated aerobic glycolysis through Na^+^K^+^ ATPase stimulation [65].

 Other inotropes such as phosphodiesterase inhibitors (e.g., milrinone) and levosimendan, a myocardium calcium sensitizer, are sometimes used in cardiogenic shock, especially in patients with previous chronic use of beta-blockers. Although their use in severe hypodynamic shock not responsive to adrenergic support seems reasonable, caution is advised in the context of refractory circulatory collapse since both have vasodilator properties and long half-life, which can worsen shock and lead to a dramatic situation [64].

### 5.7. Glucose-Insulin-Potassium Infusion

Use of a glucose-insulin-potassium (GIK) solution has been long studied in patients with acute cardiovascular disease. Theoretically, cardiac metabolism (and function) would be improved by glucose influx “forced” into the myocardium. Moreover, insulin would modulate inflammatory response and signal transduction, limiting the damage to the myocardium [66–68]. 

A randomized trial with 20201 patients with ST-Segment Elevation Myocardial Infarction showed no benefit of GIK infusion in mortality or incidence of cardiogenic shock [66]. However, it is important to note that this study was carried out in a population as a preventive measure to complications derived from ischemia, and it is hard to drawn conclusions to patients with shock from this trial, despite the large sample size.

 GIK can improve cardiac index and decrease inotropic requirement in the perioperative period of cardiac surgery [67, 68]. There are very few studies using GIK in hypodynamic inflammatory shock; however, cardiac function also seems to be improved in this situation [69]. 

 In conclusion, use of glucose-insulin-potassium solution may improve cardiac function in severe acute diseases, but no clinical benefit was found in a large randomized trial [66]. In shock scenarios, there is a clear paucity of studies of GIK solution though it seems a possible alternative in refractory hypodynamic shock [70].

### 5.8. Adjunctive Therapies

In a recent multicenter controlled trial, Schortgen et al. randomized 200 febrile patients with septic shock to aggressive fever control with external cooling or conventional treatment. The hypothesis of the trial was that lowering core temperature would increase vascular tone and decrease oxygen consumption, ameliorating shock. 

 Patients in the intervention group had lower vasopressor requirements, more shock reversal and 14-day mortality was significantly lower in the cooling group [71]. The evidence is appealing and even though more studies are necessary until fever control become state of care, control of hyperthermia may be considered in refractory shock in order to decrease vasopressor requirements. 

 Other systemic conditions that may aggravate refractory shock are hypocalcemia, hypophosphatemia, and severe acidosis. Hypocalcemia and hypophosphatemia may contribute to cardiovascular dysfunction, and electrolyte correction seems reasonable in the context of refractory shock. On the other hand, current guidelines do not recommend treatment of lactic acidosis with sodium bicarbonate if pH > 7.15 due to the risks of sodium/fluid overload and increases in lactate and PaCO_2_ [35]. However, during severe acidosis (pH < 7.15), there is scarce evidence regarding use of bicarbonate in shock patients and while therapeutic benefits are uncertain, it may be useful to correct severe hypotension in these patients. It is important to remind that correction of hypocalcemia should usually precede the use of alkaline solutions since raising pH could decrease calcium levels even more with deleterious consequences.

### 5.9. Emerging Therapies for Refractory Shock Patients

Emerging alternatives for managing refractory shock patients include extracorporeal therapies like hemofiltration, extracorporeal membrane oxygenation (ECMO), and coupled plasma filtration adsorption (CPFA).

 Small nonrandomized trials showed that some patients with refractory shock (“responders”) could benefit from short-term, high-volume isovolemic hemofiltration. However, until now there is no tool that can identify patients that will benefit from such therapy what significantly limits its clinical use [72, 73].

 Venoarterial ECMO has been long used as a bridge to refractory cardiogenic shock until myocardial recovery, revascularization, or heart transplantation. Recent data suggest also a possible role for this approach in refractory cardiac dysfunction during septic shock [74]. However, this option requires a center with ECMO expertise, what may be an important limitation in low-resource settings. 

Circulating mediators contribute to shock and organ dysfunction in inflammatory states like sepsis. CPFA aims to adsorb these mediators, impeding these harmful effects. Experimental studies suggest some hemodynamic benefit of this approach [75]. While it seems a promising alternative to treat refractory shock patients, clinical evidence is still lacking.

**Figure 1:**
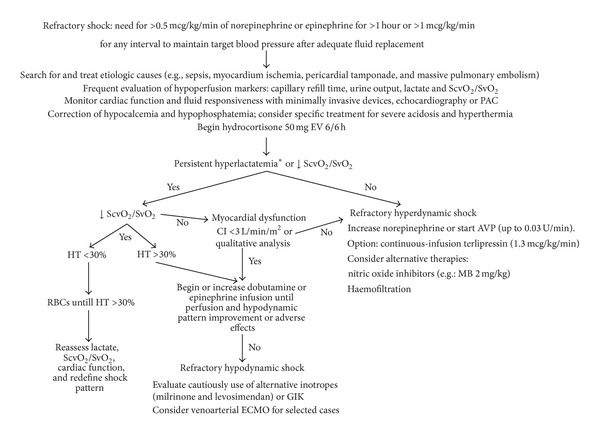
Suggested algorithm for high-dose vasopressor dependent shock. SvO_2_ = mixed venous oxygenation saturation; ScvO_2_ = superior vena cava oxygen saturation; PAC = pulmonary artery catheter; CI = cardiac index; HT = hematocrit; RBCs = red blood cell transfusions; GIK = glucose-insulin-potassium solution; AVP = arginin-vasopressin; ECMO = extracorporeal membrane oxygenation; MB = methylene blue. *See text for details.

A suggested algorithm for diagnosis and management of high-dose vasopressor dependent shock is presented in Figure 1.

## 6. Conclusion

 Although there is no consensual definition of refractory shock, the use of more than 0.5 mcg/kg/min of norepinephrine or epinephrine to maintain target blood pressure is associated with increased mortality and seems as an adequate threshold. Hemodynamic monitoring is essential since multiple mechanisms can contribute to circulatory collapse. The use of stress-dose hydrocortisone (200 mg/d) to restore vascular response to vasopressors may be beneficial in this situation. Recent data suggest that aggressive temperature control improves hemodynamics in sepsis, and measures to avoid hyperthermia should be used in the context of refractory shock. Inotrope support with epinephrine or dobutamine is often necessary to reverse cardiac dysfunction and ameliorate tissue hypoperfusion. Although arginine vasopressin infusion is frequently used in refractory shock evidence to support, this practice is still lacking. Other rescue therapies require further studies before widespread clinical use. 

## Figures and Tables

**Table 1:** Summary of studies on high-dose vasopressor dependente shock.

Study	*n*	Initial vasopressor	Severe shock definition	Outcome	%
VASST trial 2008 [3]	400	Norepinephrine	>15 mcg/min	90-day mortality	52
Park et al. 2005 [7]	20	Norepinephrine or dopamine	>0.1 mcg/kg/min or >20 mcg/kg/min	Mortality	65
Castro et al. 2008 [6]	33	Norepinephrine	>0.3 mcg/kg/min	28-day mortality	48
Benbenishty et al. 2011 [2]	48	Norepinephrine or epinephrine	>0.5 mcg/kg/min	One-year mortality	80
Dünser et al. 2003 [8]	48	Norepinephrine	>0.5 mcg/kg/min	ICU mortality	71
Torgersen et al. 2010 [9]	50	Norepinephrine	>0.6 mcg/kg/min	ICU mortality	52
DOBUPRESS study 2008 [10]	59	Norepinephrine	>0.9 mcg/kg/min	ICU mortality	68
Leone et al. 2004 [11]	17	Norepinephrine and dopamine	>2 mcg/kg/min and >25 mcg/kg/min	In-hospital mortality	47
Brown et al. 2013 [12]	443	Norepinephrine equivalent*	≥1 mcg/kg/min	90-day mortality	83
Jenkins et al. 2009 [4]	64	Norepinephrine or epinephrine	>100 mcg/min	In-hospital mortality	94
Torgersen et al. 2011 [13]	159	Norepinephrine	Need for rescue therapy with vasopressin	ICU mortality	61
Luckner et al. 2005 [14]	316	Norepinephrine	Need for rescue therapy with vasopressin	ICU mortality	51
Dünser et al. 2001 [15]	60	Norepinephrine	Need for rescue therapy with vasopressin	ICU mortality	67

*High-dose vasopressor therapy defined as dosage ≥1 mcg/kg/min of norepinephrine equivalent, calculated by adding norepinephrine equivalent infusion rates of all vasopressors.
